# Association of ideal cardiovascular health with carotid intima-media thickness (cIMT) in a young adult population

**DOI:** 10.1038/s41598-022-13994-5

**Published:** 2022-06-16

**Authors:** Vajihe Chavoshi, Maryam Barzin, Amir Ebadinejad, Pooneh Dehghan, Amin Momeni Moghaddam, Maryam Mahdavi, Farzad Hadaegh, Mahtab Niroomand, Majid Valizadeh, Fereidoun Azizi, Parvin Mirmiran, Farhad Hosseinpanah

**Affiliations:** 1 Obesity Research Center, Research Institute for Endocrine Sciences, Shahid Beheshti University of Medical Sciences, Tehran, Iran; 2 Imaging Department, Taleghani Hospital, Shahid Beheshti University of Medical Sciences, Tehran, Iran; 3 Prevention of Metabolic Disorders Research Center, Research Institute for Endocrine Sciences, Shahid Beheshti University of Medical Sciences, Tehran, Iran; 4 Endocrinology Division, Department of Internal Medicine, Shahid Beheshti University of Medical Sciences, Tehran, Iran; 5 Endocrine Research Center, Research Institute for Endocrine Sciences, Shahid Beheshti University of Medical Sciences, Tehran, Iran; 6 Nutrition and Endocrine Research Center, Research Institute for Endocrine Sciences, Shahid Beheshti University of Medical Sciences, Tehran, Iran

**Keywords:** Cardiology, Risk factors, Public health

## Abstract

Ideal cardiovascular health (CVH) is associated with a lower risk of developing cardiovascular diseases. This study aims to investigate the association of CVH metrics with carotid intima-media thickness (cIMT) as a marker of subclinical atherosclerosis in young adults. A cross-sectional study was performed on 1295 adults, average age of 29.7 ± 4.0 years, selected from the participants of the Tehran Lipid and Glucose Study. The participants were divided into three groups based on the overall CVH score: ideal, intermediate, and poor CVH. Multivariate-adjusted linear regression was used to determine the association of the CVH score with cIMT. Multivariate-adjusted odds ratios (ORs) were calculated for high cIMT (≥ 95% percentile). Also, the independent effects of each ideal CVH metric on cIMT were analyzed. The prevalence of ideal CVH was 6.4% in men and 12.4% in women, and mean cIMT was obtained 0.53 ± 0.09 mm in men and 0.57 ± 0.08 mm in women. A 1-point increase of the CVH score in men and women was associated with a cIMT decrease of 0.009 and 0.011 mm (men: Beta [SE] = − 0.009 [0.003]; women: − 0.011 [0.007], *p* < 0.001), rendering the ORs of 0.66 and 0.70 for having a high cIMT (≥ 95% percentile), respectively. Ideal blood pressure in both sexes and body mass index in women had significant inverse association with cIMT. There was an inverse graded association between the CVH score and cIMT among young adults, indicating that ideal CVH metrics were associated with better vascular health in this population.

## Introduction

Cardiovascular diseases (CVD) are the leading causes of global mortality, with approximately 17.8 million deaths reported due to diseases like coronary heart disease and stroke in just 2017^1^. More than three-quarters of CVD-related deaths occur in low- and middle-income countries^2^. The American Heart Association presented the concept of ideal cardiovascular health (CVH) in 2010, aiming to decrease CVD-related mortality by 20% in the following decade^3,4^. Cardiovascular health metrics include seven items, of which four are related to health behaviors (i.e., the smoking status, body mass index, physical activity, and diet), with the other three being important health indicators (i.e., total cholesterol, blood pressure, and fasting plasma glucose (FPG). According to studies, ideal CVH is associated with a lower CVD-related mortality and morbidity and better cardiovascular outcomes^5–7^.

Beyond traditional risk factors, subclinical atherosclerosis is an important predictor of CVD^8,9^. Carotid intima-media thickness (cIMT) is a common ultrasound-based measurement of arterial wall thickness used to evaluate atherosclerosis^10^. This parameter has been shown to predict the risk of atherosclerotic plaque formation and CVD development^11,12^. The gradual accumulation of symptom-free plaques, a process beginning at a young age, finally leads to symptomatic atherosclerosis^13^. The long duration of this preclinical phase requires detection and examination of vascular pathologic processes during early stages, which can decrease the morbidity and mortality associated with cardiovascular diseases in high-risk patients^14^. Due to the low incidence of CVD events in the young, it is difficult to establish a direct relationship between CVH and the incidence of CVDs, so it is better to seek for a surrogate diagnostic and predictive marker for CVDs in young adults. Numerous epidemiological studies have shown an association between ideal CVH and a reduction in the risk of the subclinical cardiovascular diseases characterized by changes in cIMT^6,15–18^. Evaluating the seven routine CVH metrics in Iranian men and women in the Tehran Lipid and Glucose Study (TLGS) indicated a low prevalence of ideal CVH in the adult population^19^. Therefore, it is essential to investigate a potential relationship between CVH metrics and the incidence of subclinical atherosclerosis in young adults to prevent cardiovascular events. To our knowledge, there is no study on the sex-stratified relationship between CVH metrics and cIMT in young adults, particularly in the Middle East and North Africa (MENA) region where people show a less favorable metabolic health status^20^.

This population-based study aimed to assess the association of the overall CVH score and each of the seven CVH metrics with the risk of developing subclinical atherosclerosis, defined by increased cIMT, among Iranian young adults enrolled in the framework of the TLGS.

## Material and methods

### Study participants and design

**Figure 1:**
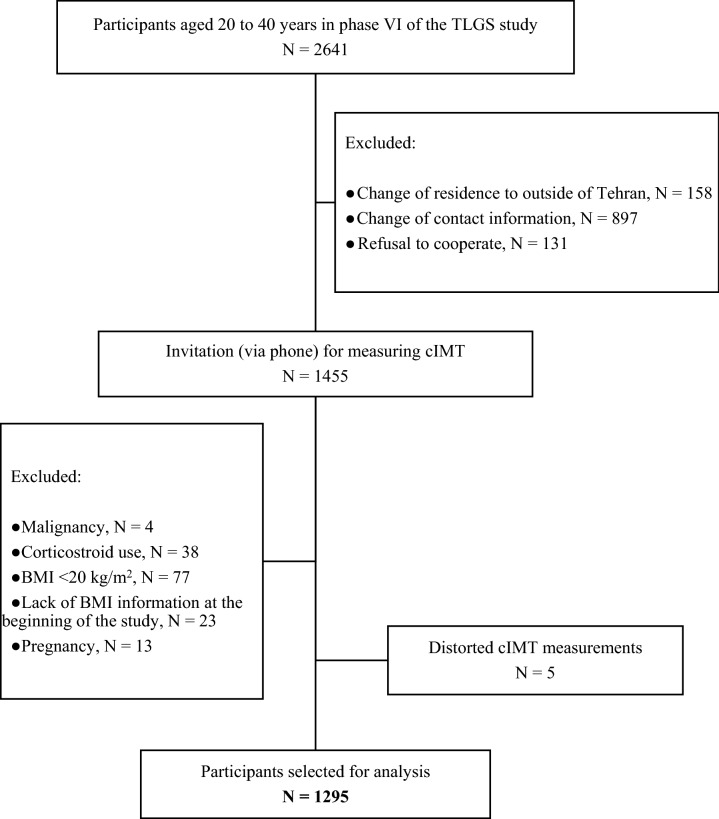
The flow chart of the study population.

In this cross-sectional study, we used the data available from the TLGS, a cohort study initiated in 1998 aiming to identify the risk factors of non-communicable diseases in Tehran urban populations. Participant recruitment in the TLGS was conducted in two phases; the first was from January 31, 1999 to July 3, 2001, and the second from October 20, 2002 to September 22, 2005. Data collection is ongoing, planned to continue for at least 20 years according to its triennial design (third phase: 2005–2008; fourth phase: 2009–2011; fifth phase: 2012–2015; and sixth phase: 2015–2018), with an average of 73% of the participants available during each phase. The details of this population-based study have been reported elsewhere^21^. In the present study, we used the data collected during the phase VI (2015–2018) of the TLGS. Among 20–40-year-old subjects undergoing routine evaluations in phase VI (n = 2641), a number of the participants were recruited for cIMT measurement (n = 1455). After excluding those with a body mass index (BMI) of < 20 kg/m^2^ at the baseline (n = 77), the subjects using corticosteroids (n = 38), pregnant women (n = 13), those with a history of malignancies (n = 4), and individuals with distorted cIMT measurement (n = 5), a total of 1295 participants were recruited for the current study (Fig. 1).

### Measurements and definitions

Using standard questionnaires, trained interviewers obtained demographic data, smoking status, dietary intake, physical activity, medical history, and drug consumption history. Also, trained personnel performed anthropometric examinations. A digital electronic weighing scale (range: 0·1–150 kg, Seca 707, Hanover, MD, the USA) was used to measure weight, which was recorded to the nearest of 100 g, while the participants were shoeless and minimally clothed. A tape meter was used to measure height in the standing position, and BMI was calculated as weight (in Kg) divided by height (in squared meters) (kg/m^2^). Duplicate measurements (15-min apart) of the systolic and diastolic blood pressures were done by a qualified physician using a standard mercury sphygmomanometer applied on the participant’s right arm while seated. The mean of the two measurements was calculated and regarded as the subject’s blood pressure. Venous blood samples were taken after 12–14 h overnight fasting, centrifuged within 30–45 min of collection, and finally analyzed at the TLGS research laboratory. Details of serum biochemistry parameters, FPG, and lipid profiles have been reported elsewhere^21^.

### Determining cIMT

The participants underwent an ultrasound using a linear 7.5–10 MHz transducer (Samsung Medison SonoAceR3 Ultrasound, South Korea). Two radiologists performed the examinations in the supine position with the neck extended and slightly rotated to the opposite side. The initial carotid scan was performed in the transverse plane across the artery to evaluate its anatomy, locate any atherosclerotic plaque, and determine the site of maximal wall-thickening. Measurements were performed in plaque-free arterial segments fulfilling the optimal B-mode imaging criteria as described below. A clear vision of the far arterial wall interface with a complete anechoic luminal content was considered an optimal greyscale carotid artery image and saved for cIMT measurement. The IMT was defined as a hypoechoic band between the arterial wall’s echogenic intimal and adventitial surfaces. The distance between the leading edge of the first and second echogenic lines of the far walls of the common carotid artery’s distal segment on both sides was measured at three locations (carotid bulb and one centimeter proximal to it), and the average was regarded as the final value on each side. It has been shown that the carotid artery’s far wall IMT measurements are more reliable than that of the near wall. In our study, IMT measurements in the left common carotid artery (LCCA) showed less inter- and intra-observer variations and were more congruent with laboratory tests’ results, so measurements on this side were chosen for analysis. The rate of agreement between the two radiologists in terms of cIMT measurements was evaluated using the inter-class correlation coefficient (ICC), and their 95% confidence intervals were calculated using SPSS statistical package software version 20 based on the two-way mixed-effects model. Accordingly, ICC was obtained as 0.79 with a 95% confidence interval of 0.55–0.90. The ICC is a value between 0 and 1, where values between 0.75 and 0.9 indicate good reliability^22^. Moreover, the mean (SD) difference of between-rate ICC was 0.08 (0.12).

### Determining CVH

We used the modified American Heart Association (AHA) 2020 Impact Goals^3^, defining three categories for CVH metrics; ideal, intermediate, and poor (Table 1). The smoking status was subdivided to the groups of never smoking, abstinence for more than 12 months, abstinence for less than 12 months, and daily or occasionally smoking, which were attributed to ideal, intermediate, and poor status, respectively. A checklist for dietary habits, a qualitative Food Frequency Questionnaire (FFQ), and two 24-h dietary recall scales were used to assess the dietary status. The validity and reliability of the Persian translated version of FFQ for evaluating the food intake status of TLGS participants have already been confirmed^3,23^. In the present study, after excluding individuals with an extreme energy intake (± 3SD), five AHA’s ideal CV health components were used to calculate the participants’ dietary scores. The Modifiable Activity Questionnaire (MAQ) was used to assess physical activity. The validity and reliability of the Persian translated version of MAQ have been confirmed for evaluating the physical activity of TLGS participants^24^. Instead of physical activity duration, we used the average metabolic equivalent task (METs) score to define physical activity. The corresponding metrics for BMI were ideal (< 25.0 kg/m^2^), intermediate (25.0–29.9 kg/m^2^), and poor (≥ 30.0 kg/m^2^). The subgroup classification of blood pressure, total cholesterol, and FPG has been illustrated in Table 1.

**Table 1:** The modified American Heart Association 2020 impact goals of ideal, intermediate, and poor CVH.

Metrics	Ideal	Intermediate	Poor
Fasting plasma glucose	< 100 mg/dL without medication	100–125 mg/dL or treated to ideal levels	≥ 126 mg/dL
Blood pressure	< 120/ < 80 mm Hg without medication	SBP 120–139 mm Hg or DBP 80–89 mm Hg or treated to ideal levels	SBP ≥ 140 or DBP ≥ 90 mm Hg
Current smoking	Never or quit ≥ 12 months	Former ≤ 12 months	Yes
Body mass index	< 25 kg/m^2^	25–29.9 kg/m^2^	≥ 30 kg/m^2^
Total cholesterol	< 200 mg/dL without medication	200–239 mg/dL or treated to ideal levels	≥ 240 mg/dL
Physical activity	≥ 1500 METs min/week	600–1500 METs min/week	< 600 METs min/week
Healthy diet score*	4–5 components	2–3 components	0–1 components

*CVH* cardiovascular health, *METs* metabolic equivalent tasks, *SBP* systolic blood pressure, *DBP* diastolic blood pressure.

*Fruits and vegetables ≥ 4.5 cups/day; fish ≥ 2 3.5-oz servings/week (preferably oily fish); fiber-rich whole grains ≥ 3 1-oz-equivalent servings/day; sodium < 1500 mg/day; sugar-sweetened beverages ≤ 450 kcal (36 oz)/week. Dietary recommendations are scaled according to a 2000-kcal/d diet.

(Circulation 2010, 121:586–613).

In the first step, we recorded each metric as a binary variable, assigning a score of 1 to the ideal category versus 0 for the poor or intermediate category, to calculate the overall CVH score. An overall CVH score was calculated by summing up the variables defined and categorized as ideal (score of 6–7), intermediate (score of 3–5), or poor (score of < 2).

### Statistical analysis

The number of ideal CVH metrics was described for the participants. Normally distributed and skewed continuous variables were presented as mean ± SD and median (IQ 25–75), respectively. Categorical variables were reported as frequencies (%). The three groups of the overall CVH score (i.e., poor, intermediate, ideal) were compared using the one-way analysis of variance (ANOVA) for normally-distributed variables, the non-parametric Kruskal–Wallis test for skewed variables, and the Chi-squared test for categorical variables. Multiple linear regression was used to assess the independent effect of ideal CVH on cIMT, adjusted for age (years), family history of premature CVD (reference: no), and educational years (reference: > 12 years). Odds for having a significantly high cIMT (cIMT > 95 percentile) were estimated using logistic regression models. The multiple imputations by chained equations (MICE) method was employed to handle missing values. All analyses were performed in STATA version 14 SE (STATA Inc., TX, the USA) regarding a two-tailed *P* value of < 0.05 as statistically significant.

### Ethical approval and consent to participate

Ethical approval for the TLGS study was obtained from the Ethics Committee of the Research Institute for Endocrine Sciences, Shahid Beheshti University of Medical Sciences. All the participants provided written informed consent. All the methods were carried out in accordance with relevant guidelines and regulations. Approval for undertaking the current project was also obtained from the Research Institute for Endocrine Sciences, Shahid Beheshti University of Medical Sciences, Tehran, Iran (IR.SBMU.MSP.REC.1399.759).

## Results

**Figure 2:**
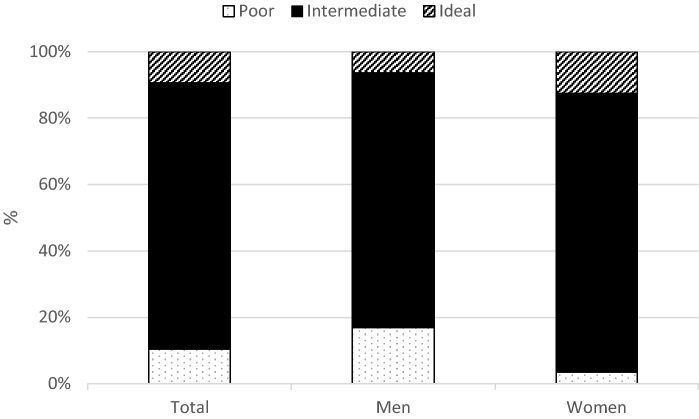
The overall CVH score among men and women. The number of participants with ideal, intermediate, and poor CVH scores was significantly different between men and women (*P *value < 0.001).

Out of 2643 young adults participating in TLGS phase VI, 753 subjects did not enter the study because they had no cIMT records. Supplemental Table 1 displays the characteristics of these individuals, as well as those included in the study (i.e., young adults with a higher proportion of men and fairly educated). The study participants’ (n = 1295) mean age was 29.7 ± 4.0 years, and 51.7% of them were men. The means of cIMT in men and women were 0.53 ± 0.09 and 0.57 ± 0.08 mm, respectively (*P* < 0.001). Male and female participants showed significant differences in BMI, blood pressure, the diet score, serum cholesterol level, FPG, and physical activity. Regarding the overall CVH score, the participants were divided into three groups. Most of the participants had intermediate CVH (n = 1039, 80.2%), followed by the poor (n = 135, 10.4%) and ideal (n = 121, 9.3%) CVH categories. More females showed an ideal CVH score than men (6.4 vs. 12.4%, *P* < 0.001). Also, the frequencies of the intermediate and poor CVH groups were significantly different between men and women (Fig. 2).

Baseline characteristics stratified according to the overall CVH score and sex have been shown in Table 2. Compared to those with low health scores, men with high CVH scores were younger; however, no such difference was observed among women. Lower values for BMI, blood pressure, total cholesterol, FPG, and the diet score were significantly associated with a higher CVH in both sexes. Participants with an ideal CVH score spent significantly more time doing moderate to vigorous physical activity. The mean of cIMT was significantly higher in men with a high vs. low CVH score (*P* = 0.018). The mean (SD) cIMT values for < 95 and > 95 percentiles were 0.53 (0.07) and 0.77 (0.06), respectively.

**Table 2:** Baseline characteristics according to the overall CVH score among men and women.

Variables	Total	Poor CVH	Intermediate CVH	Ideal CVH	*P *value
**Men**					
Number	670	113	514	43	–
Age, (year)	29.5 ± 4.0	30.4 ± 3.7	29.4 ± 4.0	28.5 ± 4.0	0.017
BMI, (kg/m2)	26.8 ± 4.7	30.5 ± 4.7	26.5 ± 4.3	21.7 ± 2.3	< 0.001
SBP, (mm Hg)	111.4 ± 11.6	118.9 ± 10.6	110.6 ± 10.9	100.4 ± 9.9	< 0.001
DBP, (mm Hg)	75.0 ± 9.2	81.5 ± 9.8	74.2 ± 8.4	66.9 ± 7.0	< 0.001
Total cholesterol, (mg/dl)	176.1 ± 37.3	204.5 ± 40.7	171.8 ± 34.1	153.3 ± 25.1	< 0.001
FPG, (mg/dl)	90.0 ± 11.2	97.8 ± 19.5	88.7 ± 7.8	86.2 ± 7.1	< 0.001
Current smoker, n (%)	256 (38.2)	83 (73.5)	173 (33.7)	0	< 0.001
Physical activity (METs min/week)	1411.0 (362.8–4280.8)	583.4 (0.00–1369.3)	1441.9 (412.4–4184.9)	5715.6 (2917.3–8573.5)	< 0.001
Healthy diet score	1.8 ± 0.9	1.8 ± 0.8	1.8 ± 0.8	1.7 ± 1.0	0.942
Family history of premature CVD, n (%)	24 (3.6)	5 (4.4)	18 (3.5)	1 (2.3)	0.803
Educational level > 12 years, n (%)	358 (53.4)	64 (56.6)	274 (53.3)	20 (46.5)	0.523
cIMT, (mm)	0.53 ± 0.09	0.56 ± 0.10	0.53 ± 0.09	0.52 ± 0.10	0.018
**Women**					
Number	625	22	525	78	–
Age, (year)	29.9 ± 4.0	29.5 ± 4.3	29.9 ± 4.0	29.9 ± 4.1	0.898
BMI, (kg/m2)	25.5 ± 4.9	30.9 ± 4.1	25.8 ± 4.9	21.8 ± 2.4	< 0.001
SBP, (mm Hg)	102.4 ± 11.1	117.7 ± 11.9	102.5 ± 10.7	97.8 ± 9.4	< 0.001
DBP, (mm Hg)	70.4 ± 8.8	81.1 ± 9.5	70.4 ± 8.7	66.9 ± 6.8	< 0.001
Total cholesterol, (mg/dl)	167.7 ± 29.4	195.7 ± 31.4	167.7 ± 29.6	159.9 ± 21.9	< 0.001
FPG, (mg/dl)	86.7 ± 7.4	93.3 ± 9.6	86.8 ± 7.3	83.7 ± 5.3	< 0.001
Current smoker, n (%)	9 (40.9)	9 (40.9)	62 (11.8)	1 (1.3)	< 0.001
Physical activity (METs min/week)	666.8 (256.4–1293.4)	666.8 (256.4–1293)	972.4 (357.7–2167.2)	2488.7 (1994.0–4073.9)	< 0.001
Healthy diet score	1.6 ± 0.8	1.6 ± 0.6	1.6 ± 0.8	1.7 ± 0.8	0.654
Family history of premature CVD, n (%)	21 (3.4)	1 (4.5)	16 (3.0)	4 (5.1)	0.605
Educational level > 12 years, n (%)	399 (63.8)	15 (68.2)	331 (63.0)	53 (67.9)	0.640
cIMT, (mm)	0.57 ± 0.08	0.60 ± 0.07	0.57 ± 0.08	0.56 ± 0.08	0.249

*BMI* body mass index, *SBP* systolic blood pressure, *DBP* diastolic blood pressure, *HDL* high-density lipoprotein cholesterol, *cIMT* carotid intima-media thickness, *CVD* cardiovascular disease, *FPG* fasting plasma glucose, *METs* metabolic equivalent tasks.

Data are presented as mean ± SD or n (%) except minutes of moderate or vigorous physical activity/week, which are presented as median (IQ 25–75).

Table 3 shows the independent effects of each ideal CVH metric on cIMT (in mm). In the model adjusted for age (model I), ideal blood pressure was significantly and inversely associated with cIMT in men (Beta [SE] = − 0.022 [0.007], *P* = 0.004). In women, ideal blood pressure and BMI were significantly and inversely associated with cIMT (ideal blood pressure: (− 0.032 [0.008], *P* < 0.001), ideal BMI: (− 0.030[0.007], *P* < 0.001)). A 1-point increase in the overall CVH score was also associated with the cIMT reductions of 0.009 and 0.011 mm in men and women, respectively. These observations did not change in the model II (adjusted for age, family history of premature CVD, and educational level).

**Table 3:** The independent effects of ideal cardiovascular health metrics on cIMT(in mm).

Variables	Model I		Model II	
	Beta (SE)	*P *value	Beta (SE)	*P *value
**Men**				
Ideal blood pressure	**− 0.022 (0.007)**	**0.004**	**− 0.021 (0.007)**	**0.005**
Ideal total cholesterol	− 0.006 (0.008)	0.511	− 0.006 (0.008)	0.481
Ideal FPG	− 0.018 (0.012)	0.132	− 0.018 (0.012)	0.135
Nonsmoking	− 0.006 (0.007)	0.467	− 0.005 (0.007)	0.484
Ideal BMI	− 0.009 (0.007)	0.249	− 0.009 (0.007)	0.262
Ideal diet	0.012 (0.029)	0.695	0.011 (0.029)	0.707
Ideal physical activity	− 0.014 (0.007)	0.067	− 0.015 (0.007)	0.052
CVH Score	**− 0.009 (0.003)**	**0.002**	**− 0.009 (0.003)**	**0.002**
**Women**				
Ideal blood pressure	**− 0.032 (0.008)**	**< 0.001**	**− 0.032 (0.008)**	**< 0.001**
Ideal total cholesterol	0.009(0.010)	0.367	0.010 (0.010)	0.355
Ideal FPG	− 0.028 (0.017)	0.108	− 0.028 (0.017)	0.111
Nonsmoking	0.008 (0.011)	0.457	0.008 (0.011)	0.454
Ideal BMI	**− 0.030 (0.007)**	**< 0.001**	**− 0.030 (0.007)**	**< 0.001**
Ideal diet	0.044 (0.028)	0.118	0.044 (0.028)	0.120
Ideal physical activity	− 0.001 (0.007)	0.842	− 0.001 (0.007)	0.847
CVH Score	**− 0.011 (0.003)**	**0.002**	**− 0.011 (0.007)**	**0.002**

*BMI* body mass index, *CVH* cardiovascular health, *CVD* cardiovascular disease, *FPG* fasting plasma glucose, *SE* standard error.

Model I adjusted for age (years).

Model II adjusted for age (year), family history of premature CVD (references: No), and Educational level (references: > 12 years).

Significant values are in bold.

Table 4 displays the independent predictive value of each ideal CVH metric (odds ratio, 95% confidence interval) for having a high cIMT (> 95 percentile). In a fully adjusted model, two metrics inversely correlated with high cIMT in men: ideal blood pressure (OR [95%CI] = 0.32 [0.15–0.65], *P* = 0.002) and ideal physical activity (0.35 [0.16–0.76], *p* = 0. 008). In women, ideal blood pressure and BMI significantly correlated with high cIMT (OR [95%CI] = 0.30 [0.16–0.58], *P* < 0.001; 0.33 [0.16–0.67], *P* = 0.003, respectively). A 1-point increase in the overall CVH score also predicted a decrease in the probability of having a high cIMT (men: 0.66 [0.51–0.86], *P* = 0.002; women: 0.70 [0.52–0.94], *P* = 0.019).

**Table 4:** The independent effects of ideal cardiovascular health metrics on cIMT (95 percentile).

Variables	Model I		Model II	
	OR (CI)	*P *value	OR (CI)	*P *value
**Men**				
Ideal blood pressure	**0.32 (0.16–0.66)**	**0.002**	**0.32 (0.15–0.65)**	**0.002**
Ideal total cholesterol	0.54 (0.27–1.09)	0.089	0.55 (0.27–1.11)	0.099
Ideal FPG	0.74 (0.27–1.98)	0.552	0.73 (0.27–1.95)	0.530
Nonsmoking	1.45 (0.70–3.01)	0.311	1.45 (0.70–3.01)	0.315
Ideal BMI	0.74 (0.33–1.51)	0.382	0.70 (0.33–1.5)	0.367
Ideal diet	–	–	–	–
Ideal physical activity	**0.35 (0.16–0.76)**	**0.008**	**0.35 (0.16–0.76)**	**0.008**
CVH score	**0.66 (0.51–0.86)**	**0.002**	**0.66 (0.51–0.86)**	**0.002**
**Women**				
Ideal blood pressure	**0.31 (0.16–0.59)**	**< 0.001**	**0.30 (0.16–0.58)**	**< 0.001**
Ideal total cholesterol	2.19 (0.66–7.28)	0.197	2.22 (0.67–7.36)	0.191
Ideal FPG	0.41 (0.13–1.25)	0.120	0.40 (0.13–1.23)	0.111
Nonsmoking	1.84 (0.55–6.12)	0.316	1.87 (0.56–6.20)	0.307
Ideal BMI	**0.34 (0.17–0.69)**	**0.003**	**0.33 (0.16–0.67)**	**0.003**
Ideal diet	3.39 (0.69–16.5)	0.131	3.41 (0.69–16.7)	0.130
Ideal physical activity	1.02 (0.54–1.90)	0.950	1.02 (0.55–1.92)	0.930
CVH score	**0.70 (0.53–0.94)**	**0.020**	**0.70 (0.52–0.94)**	**0.019**

*BMI* body mass index, *CVH* cardiovascular health, *CVD* cardiovascular disease, *FPG* fasting plasma glucose, *CI* confidence interval.

Model I adjusted for age (years).

Model II adjusted for age (year), family history of premature CVD (references: No), and Educational level (references: > 12 years).

Significant values are in bold.

## Discussion

In this population-based study conducted in the framework of the TLGS, the association of the overall CVH score and each of the seven CVH metrics with cIMT was evaluated. Participants in this study had a mean age of 30 years, and the prevalence of ideal CVH among them was 9.3%. Our results showed that a 1-point increase in the CVH score was associated with cIMT reductions of 0.009 and 0.011 mm in men and women, respectively. Also, an elevation in the CVH score predicted a decrease in the probability of having a high cIMT (> 95 percentile). Blood pressure in both sexes and BMI in women were significantly and inversely associated with cIMT. Also, having an ideal blood pressure (in both sexes), ideal BMI (in women), and ideal physical activity (in men) are associated with developing high cIMT.

The AHA introduced seven CVH metrics in the past decade to predict cardiovascular events^3^. Studies show that an ideal CVH correlates with better cardiovascular outcomes^5,7^. Obtaining an ideal CVH is important globally, especially in middle- and low-income countries^20^. In our study, the prevalence of ideal CVH among young adults was 6.4% in men and 12.4% in women. The prevalence of ideal CVH varies among populations, depending on age and gender distribution and geographic locations^25^. A systematic review of 88 studies reported that the prevalence of having five or more ideal CVH metrics was 19.6% (95% CI: 15.2–23.9%), and having a poor CVH status was about twice more prevalent in the elderly than in the young population^25^. A low prevalence (0.3–4%) of ≥ 6 ideal CVH metrics has been reported in developing countries^5^. Similarly, in the STEPwise study in Iran, although the prevalence of ideal CVH metrics among the population aged 20–65 years old reached about 7.2% in 2011, it again decreased to < 4% in 2016^26^. The metabolic health status of the people living in the MENA region is unsatisfactory due to physical inactivity and unhealthy diets; obesity is seen in more than one in every three women in most countries of the region^20^. Atherosclerosis begins in childhood and progresses during adolescence and young adulthood^27^. The prevalence and progression of fatty streaks and clinically significant lesions increase considerably during the 15–34-year age span^27^. Therefore, this age group should be under focus for examining the relationship between cardiometabolic health and subclinical atherosclerosis to prevent disease progression.

The results of our study supported earlier reports demonstrating an inverse relationship between the CVH score and cIMT^15–18^. The cIMT is a subclinical marker of atherosclerosis, predicting predisposition to cardiovascular diseases^5,11^. We found that a 1-point increase in the CVH score was associated with a cIMT decline of 0.009 mm in men and 0.011 mm in women. Also, improved CVH decreased the probability of having a high cIMT after adjustments for age and sex. Nevertheless, the association of the CVH score with cIMT did not change after further adjustments for the family history of premature CVD and educational level. The age range of our participants was between 20 and 40 years old. To our knowledge, there is no study examining the sex-stratified relationship between CVH and cIMT in young adults. We found only one cross-sectional study on a young adult population, in which five different cohorts of western populations were assessed, reporting that cIMT was 0.006 mm (95% CI: 0.012–0.003 mm) thinner for each additional CVH score^16^. Other studies on adults investigating the association between the CVH score and cIMT in Spain, the USA, and Africa revealed that a 1-point increase in the CVH score was associated with cIMT reductions of 0.011, 0.04, and 0.005 mm, respectively^15,17,18^. Santos et al*.* showed that each unit increase in cIMT increased CVH in men and women by 0.009 and 0.006, respectively. This slight difference between the two sexes compared to our study may be due to our population being younger. We found only one longitudinal study conducted in China evaluating the association between CVH metrics and cIMT. Wang et al.^28^, after excluding individuals with elevated cIMT at the baseline, examined the association of CVH metrics with cIMT changes over approximately four years and showed that the CVH score significantly and inversely correlated with the risk of developing subclinical atherosclerosis.

In our study, ideal blood pressure (in both sexes) and ideal BMI (in women only) had a significant inverse association with cIMT. Similarly, Nonterah et al.^15^ demonstrated an inverse association between the same ideal CVH metrics and cIMT in populations from four African countries. On the other hand, Oikonen et al.^16^ reported that an ideal status for each of blood pressure, BMI, cholesterol, and diet was independently and inversely associated with cIMT, whereas physical activity was directly associated with cIMT. According to these findings, each of the seven CVH metrics seems to have variable impacts on cIMT, which should be considered when evaluating the effectiveness of each metric.

The findings of this report are subjected to at least two limitations. First, because cIMT was not measured for all participants in the phase VI of the TLGS, we could not rule out the possibility of selection bias for younger individuals, men, and those with higher educational levels. However, this deviation was not clinically significant evidenced by a narrow age range of the participants (the means of age were 29.7 and 31.2 years in the included and excluded individuals, respectively). Second, it should be kept in mind that the inverse associations observed between ideal CVH metrics and cIMT were based on cross-sectional data, precluding the analysis of cause-effect associations. As the main strength, this study is the first population-based report on the sex-stratified association between CVH metrics and cIMT in a young adult population. Also, all CVH metrics were measured by trained individuals instead of relying on self-reports.

In conclusion, in this population-based study on young adults, the prevalence of having an ideal CVH score was 6.4% in men and 12.4% in women. An inverse graded association was observed between the CVH score and cIMT (i.e., a lower CVH score predicting a higher cIMT). Moreover, cIMT was significantly and inversely associated with ideal blood pressure and BMI. Future studies with larger sample sizes are suggested to investigate the relationship of ideal CVH metrics and cIMT with other surrogate markers of subclinical atherosclerosis, particularly in the MENA region. It is also necessary to conduct longitudinal studies to evaluate cIMT changes over time and assess its relationship with ideal CVH metrics, adjusting for the weight of each of the seven CVH metrics on cIMT.

## Supplementary Information

Supplementary Information.

## Data Availability

The datasets used and analyzed during the current study are available from the corresponding author on reasonable request.
