# Supplementary material for: Association of ideal cardiovascular health with carotid intima-media thickness (cIMT) in a young adult population
Source: Sci Rep. 2022 Jun 16;12:10056. doi: 10.1038/s41598-022-13994-5 (PMC9203712; doi:10.1038/s41598-022-13994-5)
Supplement: Supplementary file 1 — Supplementary Information. [file 41598_2022_13994_MOESM1_ESM.docx]

| Supplementary Table 1. Comparison of baseline characteristics of included and non-included participants in the study. | | | |
| --- | --- | --- | --- |
|  | Included | Non-included | p-value |
| Number | 1295 | 753 | - |
| Age, (year) | 29.7 ± 4.0 | 31.2 ± 10.8 | <0.001 |
| Female, n (%) | 625 (48.3) | 460 (61.1) | <0.001 |
| Weight (kg) | 74.9 ± 17.4 | 73.3 ± 17.2 | 0.054 |
| Body mass index, (kg/m2) | 26.2 ± 4.8 | 26.4 ± 5.2 | 0.253 |
| Married, n (%) | 545 (49.1) | 404 (53.7) | 0.053 |
| Educational level > 12 years, n (%) | 757 (58.5) | 391 (52.0) | 0.048 |
| Data are presented as mean ± SD or n (%) | | | |
